# Microbial orchestration of neuroimmune crosstalk: from homeostasis to disease

**DOI:** 10.3389/fimmu.2025.1679286

**Published:** 2025-10-29

**Authors:** Huixia Ouyang, Yang Yang, Xiuwei Zhang, Yiyao Cui, Yunlei Zhang

**Affiliations:** ^1^ Department of Respiratory and Critical Care Medicine, Central Laboratory, Translational Medicine Research Center, The Affiliated Jiangning Hospital of Nanjing Medical University, Nanjing, China; ^2^ School of Biomedical Engineering and Informatics, Nanjing Medical University, Nanjing, China; ^3^ Department of Thyroid and Breast Surgery, The Affiliated JiangNing Hospital of NanJing Medical University, Nanjing, China

**Keywords:** gut microbiota, neuro, immune, gut-brain-immune axis, neuroimmune homeostasis

## Abstract

The gut-brain-immune axis represents a paradigm shift in understanding systemic homeostasis and disease. While microbial dysbiosis is firmly linked to a spectrum of neurological and immunological disorders, a critical gap persists in our mechanistic understanding of how gut microbes precisely orchestrate the crosstalk between these two systems. This review moves beyond correlation to dissect the causative mechanisms by which microbial metabolites—including short-chain fatty acids, tryptophan derivatives, and neurotransmitters—directly modulate neuroimmune circuits. We synthesize emerging evidence delineating specific molecular circuits that govern microglial maturation, T cell differentiation, and blood–brain barrier integrity, and propose a novel framework for microbiota-mediated neuroimmune regulation. We evaluate cutting-edge microbiota-directed interventions, not merely as generic probiotics, but as precision tools to reestablish neuroimmune homeostasis, thereby outlining a roadmap for next-generation therapeutics in autoimmune, neurodegenerative, and psychiatric diseases.

## 1 Introduction

The gut microbiota exerts profound influence on both the nervous and immune systems, and growing evidence suggests that its most critical function lies in shaping their bidirectional communication (1). This tripartite interaction—the gut–brain–immune axis—has emerged as a key determinant of host homeostasis (2, 3). Yet a central question remains unresolved: through what molecular and cellular pathways does the microbiota calibrate this dynamic neuroimmune dialogue, and how does disruption of this regulation precipitate disease? Addressing this challenge requires disentangling the molecular signals that enable microbes to affect immune cell behavior and neuronal activity across distinct anatomical compartments. Emerging studies highlight metabolites, microbial antigens, and neuroactive compounds as candidate mediators of these effects (4–6), yet the precise mechanisms by which such signals modulate neurological function, educate peripheral immunity, and propagate through humoral, cellular, or neural routes remain incompletely defined (7, 8). Furthermore, whether deliberate modulation of the microbiota can rewire maladaptive neuroimmune circuits in established disease is an open and pressing question. In this review, we synthesize recent advances that illuminate the mechanistic basis of microbiota–neuroimmune communication. We highlight how microbial metabolites shape immune cell fate and neuronal integrity, and how perturbations in this dialogue contribute to conditions such as multiple sclerosis and autism spectrum disorders (ASD). By reframing the microbiota as an active conductor of neuroimmune crosstalk, we aim to establish a framework that integrates microbiology, immunology, and neuroscience, and to consider how microbial ecology might be leveraged for therapeutic innovation.

## 2 Gut microbiota

The human gut microbiota—a complex assemblage of bacteria, archaea, viruses, fungi, and other eukaryotes—acts as an endocrine and immunomodulatory organ central to host physiology (9–11). Beyond its established roles in nutrient metabolism and colonization resistance, it is now recognized as a key regulator of systemic homeostasis through the production of diverse bioactive metabolites. These metabolites constitute the primary medium of host–microbe communication, allowing the gut community to influence distal organs, including the brain (10). The functional capacity of the microbiota, encoded by its collective metagenome and exceeding that of the human genome, is often more consequential than its taxonomic composition (12). High-throughput sequencing has revealed that despite extensive inter-individual variation, core metabolic pathways are conserved (12). Among the most important products are short-chain fatty acids (SCFAs)—acetate, propionate, and butyrate—derived from dietary fiber fermentation. SCFAs not only sustain gut health locally but also act as histone deacetylase inhibitors and ligands for G-protein–coupled receptors (e.g., GPR41, GPR43, GPR109a), thereby modulating gene expression and function in immune cells and, potentially, neurons (13). Likewise, microbial metabolism of tryptophan yields aryl hydrocarbon receptor (AHR) ligands (e.g., indole derivatives) and serotonin (5-HT) precursors, which shape immune tolerance and neuroimmune interactions (14).

The spatial organization of microbial communities further contributes to functional diversity (15). Distinct niches along the gastrointestinal tract—from the small intestine to the colon—generate region-specific metabolic outputs that educate local immune populations and influence barrier integrity at both the gut–blood and blood–brain interfaces (15). These contributions depend on ecological stability. Perturbations from diet, antibiotics, or inflammation can induce dysbiosis, often marked by the loss of SCFA-producing taxa (e.g., *Faecalibacterium prausnitzii*) and the overgrowth of pathobionts (16). Such shifts alter the metabolic milieu, promote production of pro-inflammatory molecules such as lipopolysaccharide (LPS), and disrupt critical signaling pathways (17). Thus, the gut microbiota is best understood not as a static collection of species but as a dynamic, metabolically active interface that translates environmental inputs, particularly diet, into host biochemical signals (18, 19). The following sections examine how this microbial organ, through its metabolic repertoire and direct host interactions, calibrates the immune system, engages in bidirectional communication with the nervous system, and orchestrates integrated gut–brain–immune crosstalk.

## 3 Neuro-gut microbiota interactions: the gut–brain axis

Among the most intensively investigated topics in biomedical research is the bidirectional communication between the gut microbiota and the nervous system, commonly referred to as the gut–brain axis (GBA) (20, 21). This complex, multidimensional network facilitates continuous signaling between the gastrointestinal tract and the central nervous system (CNS), influencing not only gastrointestinal physiology but also mood, cognition, behavior, and overall systemic health. The GBA encompasses four interconnected components: the gut microbiota, the central and peripheral nervous systems, the neuroendocrine system, and the immune system (22). Within the CNS, regions critical to the GBA include the prefrontal cortex, limbic system, and hypothalamic–pituitary–adrenal (HPA) axis (23). The limbic system—particularly the hippocampus—serves as a central hub for emotional regulation, memory consolidation, and behavioral adaptation, integrating neural inputs from diverse brain regions (24). The peripheral arm of the GBA comprises the autonomic nervous system (ANS), including both sympathetic and parasympathetic branches, and the enteric nervous system (ENS) (25–27). The ENS, embedded within the gastrointestinal wall, functions semi-autonomously to regulate gut motility, secretion, and local reflexes. It maintains direct anatomical and functional connections to the brain via the vagus nerve, dorsal root ganglia, and nodose ganglia, thereby constituting the structural basis for gut–brain feedback loops (28). The ANS modulates gut activity in response to systemic cues: sympathetic activation during stress suppresses motility and modulates immune responses, whereas parasympathetic input, primarily through the vagus nerve, supports digestion, mucosal immunity, and microbial equilibrium under homeostatic conditions (29). Through these integrated pathways, the gut microbiota exerts influence on neural circuits by producing neuroactive metabolites, modulating neurotransmitter availability, and shaping immune signaling. Conversely, central nervous outputs regulate microbial composition and activity via neuroendocrine signaling and autonomic efferents. This intricate bidirectional crosstalk underlies the pathophysiology of numerous disorders, including depression, anxiety, irritable bowel syndrome (IBS), and neurodevelopmental conditions (30, 31).

### 3.1 Neurobiological pathways linking the CNS and gut microbiota

**Figure 1:**
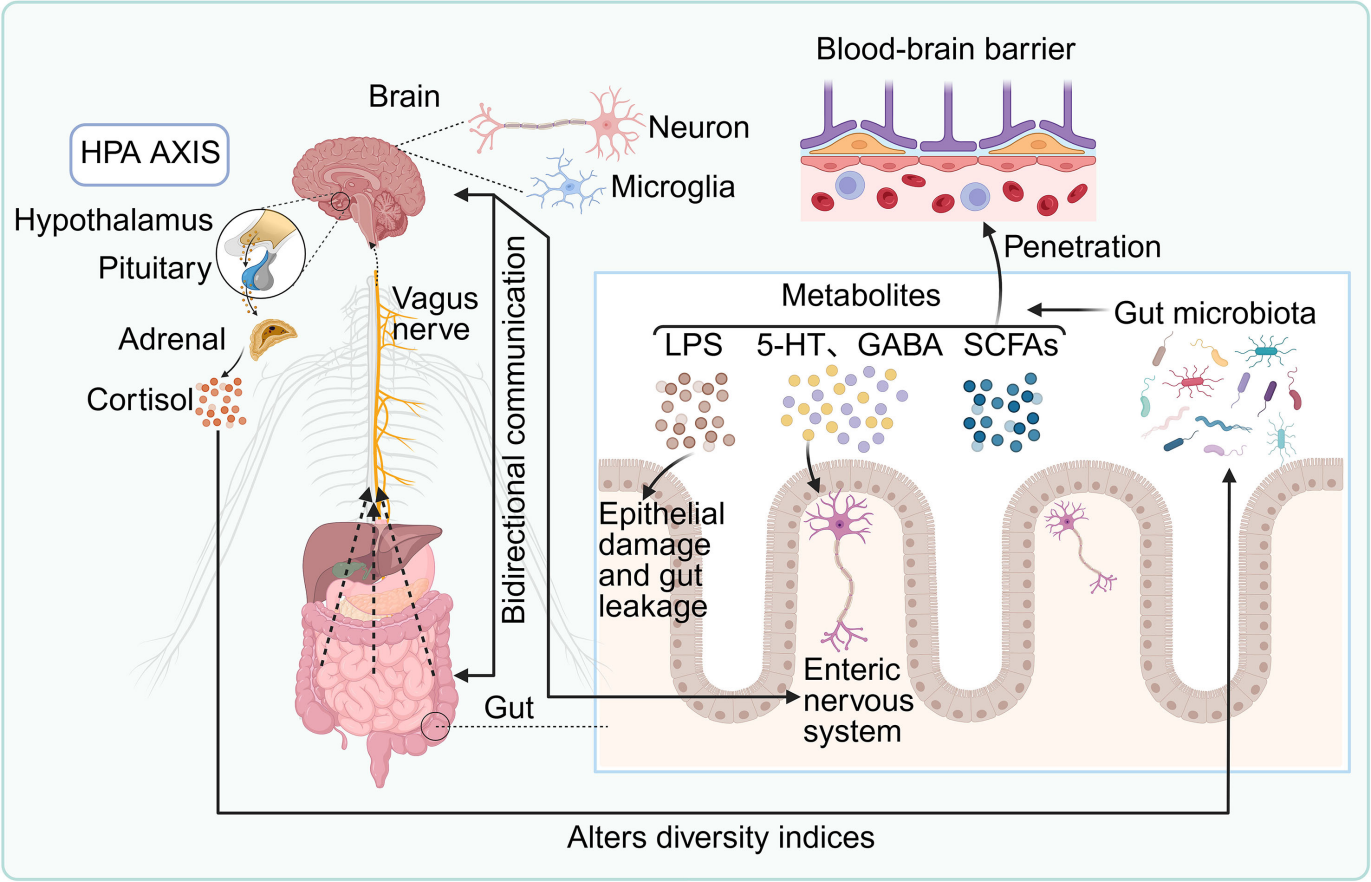
Bidirectional communication between the gut microbiota and the central nervous system (CNS). Microbial metabolites—including lipopolysaccharide (LPS), serotonin (5-HT), γ-aminobutyric acid (GABA), and short-chain fatty acids (SCFAs)—can cross the blood–brain barrier (BBB) to modulate neuronal and microglial function, thereby influencing brain physiology. The gut microbiota also interacts with the enteric nervous system (ENS), regulating epithelial barrier integrity and contributing to intestinal permeability. The hypothalamic–pituitary–adrenal (HPA) axis communicates with the gut directly via the vagus nerve and indirectly through cortisol secretion, which in turn modulates microbial composition. This reciprocal signaling network within the gut–brain axis (GBA) is critical for neuroimmune homeostasis and plays a central role in the pathogenesis and progression of neuroimmune disorders.

Bidirectional communication between the CNS and gut microbiota is mediated through four principal, interconnected pathways: neural circuits, intestinal barrier (IB) integrity and inflammation, neuroendocrine signaling, and microbial modulation of neurotransmitters (**Figure 1**). Neural signaling constitutes the most direct and evolutionarily conserved mode of interaction (32–34). The vagus nerve, a major afferent–efferent conduit of the GBA, senses microbial metabolites—including SCFAs, tryptophan derivatives, and secondary bile acids—via its peripheral terminals, transmitting these signals to brain regions implicated in emotion and homeostasis (35, 36). Vagal afferents serve as the primary gut-to-brain channel, while efferent vagal activity regulates intestinal immune tone through the cholinergic anti-inflammatory reflex, suppressing pro-inflammatory cytokines such as TNF-α and modulating macrophage responses. Enhanced vagal tone has been associated with changes in appetite regulation, mood, and systemic inflammation (37). Complementing vagal pathways, the ENS—comprising 200–600 million neurons and extensive enteric glia—functions semi-autonomously to control gut motility, secretion, and mucosal immunity, while maintaining reciprocal communication with the CNS via spinal and vagal afferents (38–40).

The IB–inflammation axis serves as a second key communication route. Dysbiosis or pathogen invasion can compromise tight junction integrity through downregulation of occludin, claudins, and zonula occludens-1, permitting translocation of microbial components into the lamina propria (41). Recognition of pathogen-associated molecular patterns, such as LPS, by pattern recognition receptors activates innate immune responses, triggering local and systemic inflammation (42). Immune signals then reach the CNS via humoral and neural pathways, contributing to neuroinflammation and altered brain function (43, 44).

The neuroendocrine axis, notably the HPA axis, represents the third mode of interaction. In response to stress, enteroendocrine cells and ENS neurons release hormones and neuropeptides—including corticotropin-releasing hormone (CRH), substance P, and various gut peptides—that activate the HPA axis through vagal and circulatory routes (45, 46). Cortisol, released by the adrenal cortex, modulates IB integrity, immune cell activity, and microbiota composition, forming a feedback loop that influences metabolism, immune tolerance, and neurobehavioral outcomes (47).

Finally, microbial modulation of neurotransmitters constitutes the fourth core pathway. The gut microbiota produces or modulates numerous neuroactive compounds, including catecholamines (dopamine, norepinephrine (NE), epinephrine), 5-HT, GABA, and histamine (4, 48, 49). Dopamine, primarily synthesized in the CNS, is influenced by gut microbes and local catecholamine production, impacting vascular tone, reward pathways, and stress responses in a dose-dependent manner (50, 51). Sympathetic neurons and the adrenal medulla synthesize NE and epinephrine, which, as key mediators of the ‘fight-or-flight’ response, potently suppress intestinal motility and reduce perfusion via vasoconstriction, while also altering blood flow distribution to modulate nutrient absorption and immune activity, effectively diverting energy away from digestion during stress (31, 49). Approximately 90% of 5-HT is produced in the gut, where microbial-derived SCFAs and tryptophan metabolites regulate its biosynthesis in enterochromaffin cells; 5-HT subsequently modulates CNS signaling via circulation and vagal pathways (52, 53). GABA, the principal inhibitory neurotransmitter in the CNS, is synthesized by *Lactobacillus* and *Bifidobacterium* through glutamate decarboxylase activity. Microbial GABA influences enteric neural function, visceral sensitivity, insulin secretion, and immune responses—including IL-6 suppression—and has been implicated in anxiety, depression, and IBS (54, 55). Histamine, generated by bacterial histidine decarboxylase (notably in *Lactobacillus* and *Escherichia coli*), modulates gut motility and immune tone via enteric H1–H4 receptors and may cross the BBB during inflammation, activating microglia and contributing to neuroimmune dysregulation (56, 57). While physiological histamine levels support arousal and memory, dysregulated microbial histamine production has been linked to migraines, IBS, and anxiety disorders (58).

Furthermore, indoleacetic acid (IAA), a tryptophan metabolite, plays regulatory role in neuroinflammation and neurodegeneration. Research indicates that *Bacteroides fragilis*, *Bacteroides thetaiotaomicron*, and *Anaerostipes hadrus* are tryptophan-metabolizing bacteria. The IAA they produce acts as a ligand for the AHR (59). By activating AHR, IAA inhibits glial cell activation and inflammatory mediator release, and suppresses the activation of the receptor for advanced glycation end products (RAGE) pathway via AHR-dependent signaling, thereby inhibiting the NF-κB signaling pathway. This reduces the expression of inflammatory cytokines (such as TNF-α, IL-1β, IL-6, and iNOS) and alleviates neuroinflammation (60). Similarly, research indicates that the synbiotic combination of *Lactobacillus suilinensis* and inulin increases indole-3-lactic acid synthesis, activates the AHR signaling pathway, suppresses the expression of pro-inflammatory factors like TNF-α, COX2, and IL-1β, reduces microglial activation, and alleviates systemic and CNS inflammatory responses (61). Additionally, studies have shown that gut microbiota alterations associated with mild cognitive impairment (MCI) in Down syndrome patients may induce systemic inflammation, thereby compromising BBB integrity. For example, increased *Bacteroides* levels correlate with elevated YKL-40 (a neuroinflammation marker), which may indirectly affect brain health by triggering immune responses and inflammatory processes (62). Collectively, these four pathways illustrate the complex, multimodal nature of CNS–microbiota communication, providing mechanistic insight into how the gut ecosystem shapes neural function and systemic health.

### 3.2 Gut microbiota regulation of neuroimmune function via BBB integrity

The BBB comprises brain microvascular endothelial cells (BECs), pericytes, basement membrane, and astrocytes. It maintains its structural and functional integrity through tight junctions and adherens junctions, effectively preventing harmful substances from entering the brain. Systemic inflammation can downregulate the expression of junctional proteins such as claudin-5, occludin, and VE-cadherin in BECs, thereby increasing BBB permeability (8). This process is precisely regulated by multiple signaling pathways, including Wnt/β-catenin, Sonic Hedgehog, and PDGF-β/PDGFRβ, and their dysregulation is closely associated with BBB disruption (63). Gut dysbiosis can induce intestinal inflammation, which in turn triggers systemic chronic inflammation. Inflammatory mediators such as IL-1β and TNF-α can enter the circulatory system through the compromised IB, disrupting BBB structure and facilitating the entry of inflammatory mediators or pathogens into brain tissue. This activates microglia, initiating neuroinflammatory responses (2).

Additionally, the gut microbiota influences the balance between Th17 cells and regulatory T cells (Tregs). For example, dysbiosis leads to excessive activation of Th17 cells, resulting in the production of pro-inflammatory factors such as IL-17A, IFN-γ, TNF-α, and IL-6 (64). These factors can upregulate inflammatory markers on BEC surfaces (e.g., VCAM-1 and ICAM-1), facilitating the transmigration of immune cells (such as Th17 and other CD4+ T cells) across the BBB into the brain, thereby exacerbating neuroinflammation and neurodegenerative diseases (65, 66).

In contrast to these pro-inflammatory mechanisms, certain gut microbiota metabolites—including SCFAs, bile acids, and H_2_S—exert beneficial effects on IB function and systemic inflammation. SCFAs possess anti-inflammatory properties, enhancing intestinal epithelial barrier integrity and suppressing systemic inflammatory responses (67). They can also cross the BBB to influence neurotransmitter levels and neuronal activity in the brain. For instance, SCFAs regulate GABAergic neurotransmission, thereby influencing mood and cognitive function (68).

### 3.3 Neuroendocrine regulation of host–microbiota interactions

In contrast to the well-studied bottom-up signaling from gut to brain, efferent pathways from the CNS back to the gut play an equally vital role in shaping the microbiota. The ANS and neuroendocrine signals allow the host to rapidly adapt to psychological and physiological stressors by altering the gut microenvironment (65, 69, 70). Activation of the sympathetic nervous system (SNS) under stress significantly influences gut function and microbial composition. NE released from sympathetic neurons can directly modulate bacterial growth, virulence, and gene expression (71). Certain pathogens, including *Escherichia coli* and *Salmonella enterica*, express adrenergic receptors that detect NE and enhance their growth and pathogenicity (72). Chronic stress-induced SNS activation promotes dysbiosis, reducing microbial diversity and depleting beneficial taxa such as *Lactobacillus*, likely through NE-mediated growth inhibition and changes in intestinal motility and mucus secretion (73, 74).

Conversely, the parasympathetic nervous system—primarily through the vagus nerve—supports a “rest-and-digest” state that favors commensal microbes. Vagal efferent activity enhances intestinal motility, stimulates epithelial secretion, and helps maintain mucosal barrier integrity (75, 76). Its cholinergic anti-inflammatory pathway further suppresses local immune activation, fostering a less inflammatory microbial environment (77). Vagotomy studies confirm that vagal signaling directly influences microbial community structure, underscoring its role in maintaining gut ecological balance (78, 79). Neuroendocrine hormones also serve as key mediators of brain-to-gut communication. CRH and glucocorticoids (cortisol in humans, corticosterone in rodents)—core components of the HPA axis—modulate gut function during stress. Glucocorticoids can inhibit the growth of certain commensals (80), compromise IB function (“leaky gut”) (81), and alter bile acid profiles, thereby applying selective pressure on the microbiota (82). Other stress hormones, including catecholamines, enter the gut lumen and may be used by bacteria as nutrients or signals, further influencing microbial ecology (83). Together, these top-down mechanisms ensure continuous adjustment of the gut microbiota by the host’s central state, forming a feedback loop in which cognitive and emotional states rapidly affect microbial composition and function, which in turn modulate brain activity.

### 3.4 Gut microbiota dysbiosis and neurological disorders

Accumulating evidence implicates gut microbiota dysbiosis in the pathogenesis of several major neurodegenerative and neuroinflammatory disorders, including Parkinson’s disease (PD), Alzheimer’s disease (AD), and MS (84). Alterations in microbial composition and metabolite profiles may affect neural health through immune activation, disruption of BBB integrity, altered neurotransmitter synthesis, and modulation of neuroinflammation and host metabolism.

#### 3.4.1 Parkinson’s disease

PD is clinically characterized by progressive motor impairments—bradykinesia, resting tremor, and rigidity—as well as non-motor symptoms such as depression, cognitive decline, and autonomic dysfunction (85). Increasing studies reveal shifts in gut microbiota composition associated with PD. Compared to healthy controls, patients exhibit reduced abundance of anti-inflammatory and SCFA-producing taxa, including *Lachnospiraceae*, *Prevotellaceae*, *Faecalibacterium*, and *Bacteroides*, alongside increased levels of *Akkermansia*, *Bifidobacterium*, and *Lactobacillus*, taxa linked to LPS and toxin production (86, 87). The observed increase in typically beneficial genera (*Akkermansia*, *Bifidobacterium*, *Lactobacillus*) is not necessarily contradictory but reflects a broader ecological shift towards a pro-inflammatory state in PD. Their association with LPS and toxin-related pathology is primarily indirect and context-dependent (88, 89). For example, *Akkermansia muciniphila* is a specialized mucin-degrader. While beneficial in homeostasis, its overgrowth can degrade the protective mucus layer. This erosion facilitates the translocation of pro-inflammatory microbial products, including LPS from other Gram-negative bacteria, into the host system, potentially triggering systemic inflammation (88, 90). *Bifidobacterium* and *Lactobacillus* are Gram-positive and do not produce LPS. Their increase may occur alongside unmeasured expansions of LPS-producing pathobionts (e.g., *Enterobacteriaceae*) (91). Thus, they serve as a marker of dysbiosis rather than a direct source of LPS (17).

This microbial imbalance compromises IB integrity, increasing gut permeability and systemic inflammation, which may exacerbate CNS dysfunction (92, 93). A hallmark of PD pathology is the misfolding and aggregation of α-synuclein (α-syn) within enteric neurons, potentially originating in the submucosal plexus or olfactory bulb and propagating to the brainstem via retrograde transport along the vagus nerve (94). Transplantation of fecal microbiota from PD patients into mice worsens α-syn aggregation and motor deficits, supporting the gut-origin hypothesis (10). Recent studies indicate that PD patients exhibit an increased abundance of *Streptococcus mutans (S. mutans)* in their gut. This bacterium utilizes uridine reductase to produce the metabolite imidazole propionate (ImP), which can cross the BBB and enter the brain. Within the brain, ImP activates the mTORC1 signaling pathway, contributing to the specific loss of dopaminergic neurons—a hallmark of PD—and consequently triggering motor dysfunction (95). Furthermore, ImP exacerbates α-syn pathology by promoting its aggregation, which further intensifies neurotoxicity. Additionally, ImP activates microglia (as evidenced by enlarged cell bodies) and induces astrocyte activation, thereby driving neuroinflammation (95). Microbial-derived SCFAs can activate neuroprotective pathways by inducing glial cell-derived neurotrophic factor and brain-derived neurotrophic factor (BDNF), while dysbiosis also affects ghrelin and other neuropeptide signaling, further modulating disease progression (96).

#### 3.4.2 Alzheimer’s disease

AD, the leading cause of dementia, is defined by extracellular β-amyloid (Aβ) plaques and intracellular neurofibrillary tangles composed of hyperphosphorylated tau protein (97). Patients with AD show distinct gut microbial alterations characterized by depletion of key SCFA-producing taxa, particularly butyrate-producing members of the families *Lachnospiraceae* and *Ruminococcaceae* (within the phylum *Bacillota*) as well as the genus *Bifidobacterium* (98). This is accompanied by an increased abundance of pro-inflammatory bacteria, including those within the phylum *Proteobacteria* (e.g., *Escherichia coli*). This dysbiosis reduces neuroprotective metabolites like butyrate and increases endotoxin release (e.g., LPS), which activates Toll-like receptor 4 (TLR4) signaling, triggering peripheral immune responses and elevating pro-inflammatory cytokines IL-6 and TNF-α (99).

These cytokines cross the BBB or signal via neural pathways to activate microglia, sustaining neuroinflammation and accelerating Aβ deposition and tau hyperphosphorylation—key drivers of neurodegeneration (100). Compared to healthy individuals, patients with AD and those with MCI exhibit higher levels of bacterial extracellular vesicles (bEVs) in their blood. These bEVs, derived from the gut microbiota, contain elevated concentrations of LPS (101). It is proposed that these LPS-bearing bEVs can traverse the BBB. Upon entering the brain, the LPS cargo activates the Piezo1 mechanosensitive channel on microglia. This Piezo1 activation triggers the classical complement cascade, specifically the C1q-C3 pathway, which leads to excessive synaptic pruning (101). This aberrant microglial activity, driven by gut microbiota-derived bEVs, represents a novel mechanism contributing to synaptic loss and neuroinflammation in AD. Gut microbes also influence neurotransmitter bioavailability (dopamine, GABA, 5-HT, acetylcholine (ACh), melatonin) and affect autophagy, oxidative stress, and glial function, all implicated in AD pathology (21).

#### 3.4.3 Multiple sclerosis

MS is an autoimmune demyelinating disorder of the CNS, shaped by genetic susceptibility, environmental factors, and immune dysregulation (102). MS patients exhibit altered gut microbial profiles compared to healthy controls, marked by reduced levels of *Bacteroides*, *Prevotella*, *Faecalibacterium*, *Lactobacillus*, and *Clostridium*, alongside increased abundance of *Pseudomonas*, *Haemophilus*, and *Mycoplasma* (103–105). Dysbiosis contributes to an imbalance between pro-inflammatory Th17 cells and anti-inflammatory Tregs. Segmented filamentous bacteria (SFB) promote Th17 differentiation and secretion of IL-17 and IL-22, exacerbating CNS inflammation, whereas SCFA-producing *Clostridia* are diminished, reducing Treg-mediated immunosuppression (66). Molecular mimicry between microbial antigens—such as from *Klebsiella pneumoniae*—and CNS myelin proteins (e.g., myelin oligodendrocyte glycoprotein) may activate autoreactive B cells (106). Microbial products like LPS increase BBB permeability and stimulate peripheral macrophages to release pro-inflammatory cytokines (5, 107). Additionally, tryptophan-derived microbial metabolites modulate microglial activation via vagal afferents, while increased BBB permeability facilitates CNS infiltration by CD4+ T cells (108–110). Collectively, these pathways underscore the multifactorial role of gut microbiota in MS pathogenesis, encompassing immune modulation, barrier disruption, and neuroinflammatory cascades.

### 3.5 Gut microbiota in neuropsychiatric disorders

Beyond classical neurodegenerative diseases, alterations of the gut microbiota are increasingly implicated in major neuropsychiatric disorders, including depression, anxiety, and ASD. In these contexts, microbial dysbiosis does not simply accompany disease but contributes to pathogenesis by reshaping systemic immunity, modulating neurotransmitter pathways, and altering neural signaling via microbial metabolites.

#### 3.5.1 Depression and anxiety

Clinical and preclinical evidence supports a bidirectional relationship between dysbiosis and mood disorders. Patients with major depressive disorder (MDD) or anxiety frequently exhibit reduced microbial richness, characterized by depletion of butyrate-producing taxa such as *Faecalibacterium* and *Coprococcus*, alongside expansion of pro-inflammatory families like *Enterobacteriaceae* (111, 112). Causality has been demonstrated by fecal microbiota transplantation (FMT) from depressed individuals into germ-free rodents, which transfers depression-like phenotypes (113). Mechanistically, dysbiosis influences neuropsychiatric states through several converging pathways. Mechanistically, gut dysbiosis contributes to neuropsychiatric disorders through converging immune, metabolic, and neural pathways. Translocation of microbial products such as LPS elevates systemic pro-inflammatory cytokines (e.g., IL-6, TNF-α), creating an inflammatory milieu that impairs hippocampal neurogenesis and reduces BDNF, both essential for mood regulation (2). In parallel, disruption of microbial communities alters tryptophan metabolism, diverting it from 5-HT synthesis toward the kynurenine pathway and producing neurotoxic metabolites that exacerbate neuroinflammation (114). Additionally, specific probiotic strains, including *Lactobacillus* and *Bifidobacterium*, exert vagus-dependent effects on the HPA axis, reducing cortisol levels and mitigating anxiety-like behaviors (115). Together, these pathways reveal how dysbiosis can both initiate and amplify the neuroimmune and neuroendocrine disturbances characteristic of depression and anxiety.

#### 3.5.2 Autism spectrum disorder

Gut microbiota disturbances are also prominent in ASD, where gastrointestinal comorbidities are frequent. Children with ASD often display increased abundance of *Clostridium* and decreased *Bifidobacterium* species (116, 117). Preclinical studies indicate a causal role: maternal immune activation induces offspring dysbiosis and ASD-like behavioral abnormalities, which can be ameliorated by probiotic administration of *Bacteroides fragilis* (116–118). Microbial metabolites emerge as critical mediators. Elevated levels of 4-ethylphenylsulfate, a microbial-derived aromatic compound, induce anxiety-like behaviors in mice (118). Similarly, propionic acid, an abundant SCFA, exerts dose-dependent effects—supporting gut physiology at low concentrations but provoking neuroinflammation and ASD-like behaviors when present in excess (119). Perturbations in bile acid metabolism and reductions in microbial-derived GABA and 5-HT precursors have also been described, linking dysbiosis to disrupted neurotransmitter homeostasis.

## 4 Gut microbiota–immune system crosstalk

Disruptions within the microbiota–immune axis have been linked to a broad array of immune-mediated disorders, including gastrointestinal infections, inflammatory bowel disease (IBD), metabolic conditions such as cardiovascular disease, diabetes, and hypertension, autoimmune diseases including rheumatoid arthritis, hypersensitivity reactions like asthma and food allergies, neuropsychiatric disorders such as anxiety, and malignancies including colorectal and hepatocellular carcinoma.

### 4.1 Microbiota-mediated immune development and homeostasis

**Figure 2:**
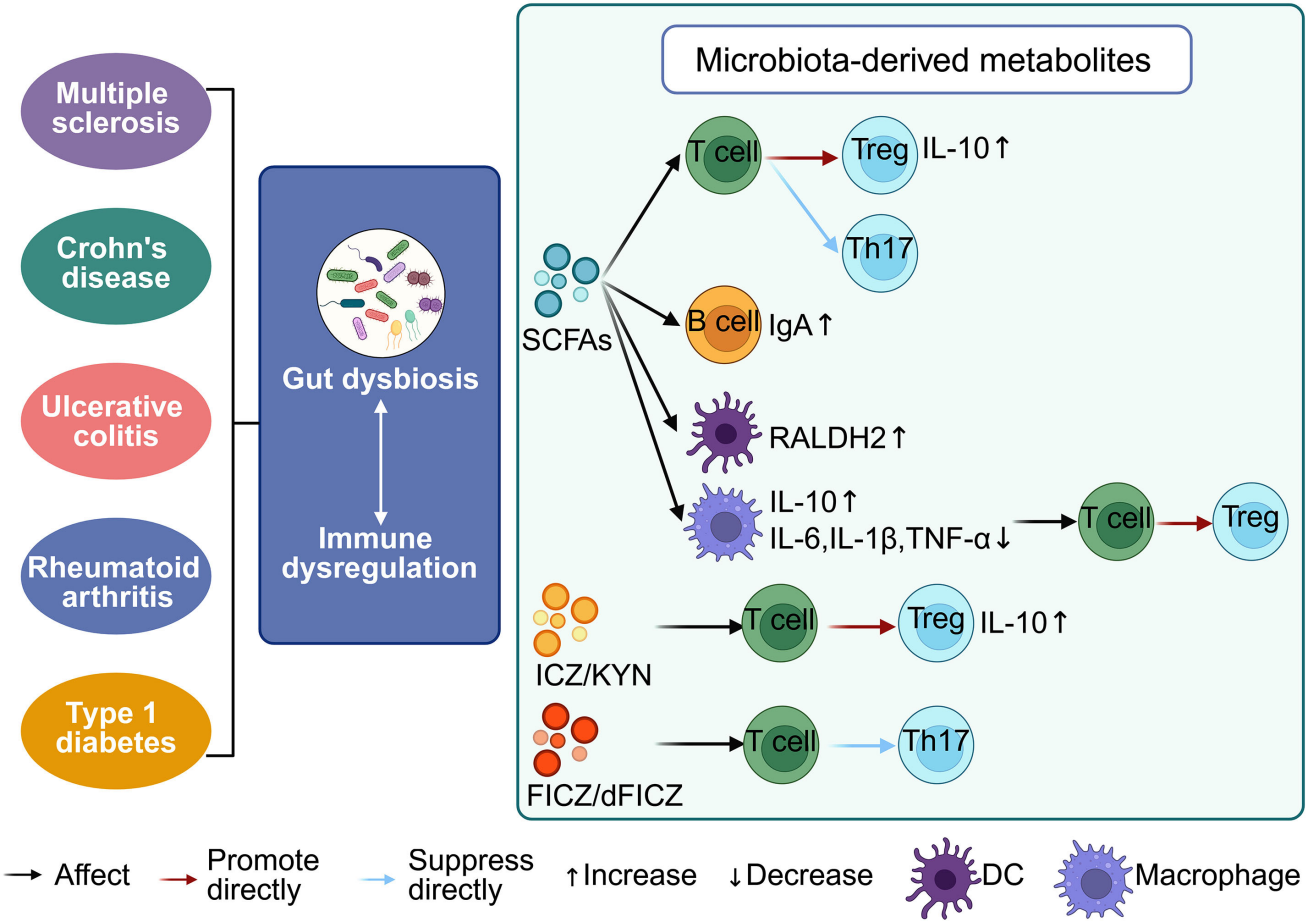
Interaction between the gut microbiota and the host immune system. Gut microbiota dysbiosis disrupts immune homeostasis, contributing to the pathogenesis of autoimmune diseases such as multiple sclerosis, Crohn’s disease, ulcerative colitis (UC), and rheumatoid arthritis. Microbial metabolites—including short-chain fatty acids (SCFAs), indole derivatives like indolo [3, 2-b] carbazole (ICZ), kynurenine (KYN), and 6-formylindolo [3, 2-b] carbazole (FICZ/dFICZ)—modulate immune cell function by influencing cytokine profiles and T cell differentiation. SCFAs suppress pro-inflammatory cytokines (IL-6, IL-1β, TNF-α), enhance anti-inflammatory IL-10 production, and promote IgA secretion by B cells. Both SCFAs and ICZ/KYN drive Treg differentiation, whereas FICZ/dFICZ inhibit Th17 cell development, underscoring the immunoregulatory potential of microbiota-derived metabolites.

Studies employing GF animal models have unequivocally demonstrated that the gut microbiota is indispensable for postnatal immune maturation (120). GF mice display profound immunodeficiencies, including impaired differentiation of Th17 cells, an imbalance in Tregs, underdeveloped Peyer’s patches, and reduced IgA production (121, 122). Colonization with defined microbial taxa rescues these defects, underscoring the causal role of microbiota in immune programming. For example, SFB promote Th17 differentiation via the IL-23/IL-1β axis, while *Bacteroides fragilis* produces polysaccharide A (PSA) that activates dendritic cells to expand Tregs and foster immune tolerance (123). Similarly, *Clostridium* species enhance Treg differentiation through SCFAs, particularly butyrate, via epigenetic mechanisms such as histone acetylation (124). Additionally, *Bacteroides* and probiotic strains like *Escherichia coli* Nissle 1917 facilitate B cell maturation and immunoglobulin class switching (IgA, IgG), supporting mucosal defense (124). These interactions occur both through direct microbe–immune cell contact and indirectly via microbial metabolites and pattern recognition receptor (PRR) signaling pathways, including Toll-like receptors and NOD-like receptors (**Figure 2**) (125). Collectively, these processes establish a finely tuned immune milieu balancing host defense and tolerance. The capacity of specific microbial strains to differentially modulate immune pathways highlights their potential as therapeutic agents. Rational probiotic design, metabolite supplementation (e.g., SCFAs), and precision microbial consortia represent promising immunomodulatory strategies for autoimmune, allergic, and inflammatory diseases.

### 4.2 Microbial metabolites as immunomodulators

Microbial-derived metabolites form a critical interface between the gut microbiota and host immunity, orchestrating immune tolerance, inflammatory responses, and cellular metabolism (126). The gut microbiota exerts profound influence on brain and neuroimmune function by modulating key neurotransmitter pathways (127). The GABAergic/Glutamatergic balance is a prime target, with microbes producing inhibitory GABA and consuming excitatory glutamate precursors, thereby regulating neuronal network activity (128). Simultaneously, microbial SCFAs enhance cholinergic function, which supports synaptic plasticity and provides potent anti-inflammatory signals via the cholinergic anti-inflammatory pathway (129). The serotonergic and dopaminergic systems are similarly modulated, as microbes supply essential precursors (tryptophan, tyrosine) and bioactive metabolites that influence the synthesis of serotonin and dopamine (53, 130). These monoamines subsequently regulate neuroimmune cells, such as microglia, and refine synaptic architecture, ultimately integrating gut-derived signals with central control of behavior, plasticity, and immunity (131). To provide a comprehensive overview of the roles of microbiota and their metabolites in neuroimmune function, we summarize key findings in **Table 1**.

**Table 1:** Microbiota and metabolites in neuroimmune function.

Microbiota or metabolite	Mechanism of action	Associated diseases	References
Butyrate	Primary energy substrate for colonocytes; reinforcement of epithelial barrier function; attenuation of intestinal inflammation via NF-κB signaling suppression	IBD, Metabolic Syndrome	(132, 133)
Acetate and Propionate	Regulation of mucosal immunity; promotion of Treg differentiation and immune tolerance	IBD, Metabolic Syndrome	(134, 135)
Tryptophan Metabolites (e.g., Indole Derivatives)	Inhibits glial cell activation and release of inflammatory mediators via AHR signaling; suppresses RAGE pathway activation, NF-κB signaling, and inflammatory cytokine expression	Neuroinflammation, Neurodegenerative Diseases	(60, 136)
GABA	Synthesis by Lactobacillus and Bifidobacterium via glutamate decarboxylase activity; regulation of enteric neural function, visceral sensitivity, insulin secretion, and immune responses	Anxiety, Depression, IBS	(54, 55)
5-HT	Production in the gut (~90%); regulation by microbial-derived SCFAs and tryptophan metabolites; modulation of CNS signaling via circulation and vagal pathways	Depression, Anxiety, IBS	(52, 53)
Dopamine	Synthesis by gut microbes such as Bacillus spp. and Escherichia spp.; modulation of immune responses and central dopamine pools	PD, Inflammatory Disorders	(137–139)
Histamine	Production by bacterial histidine decarboxylase (e.g., Lactobacillus, Escherichia coli); regulation of gut motility and immune tone via H1–H4 receptors; potential BBB penetration during inflammation	Migraines, IBS, Anxiety Disorders	(56, 140)
*Akkermansia muciniphila*	Stimulation of mucin production; modulation of epithelial renewal and differentiation; enhancement of chemical barrier	IBD, Metabolic Syndrome	(141)
*Faecalibacterium prausnitzii*	Anti-inflammatory signaling; inhibition of intestinal inflammation	IBD, Metabolic Syndrome	(142–145)
*Bifidobacterium*	Enhancement of tight junction integrity	IBD, Metabolic Syndrome	(25, 146, 147)
*Lactobacillus*	Competitive exclusion of pathogens	IBD, Metabolic Syndrome	(142–145)
*Streptococcus mutans*	Production of ImP; activation of mTORC1 signaling; loss of dopaminergic neurons and exacerbation of α-syn pathology, leading to motor dysfunction	PD	(95)
*Lactobacillus acidophilus*	Enhancement of intestinal absorption of linoleic acid; activation of peroxisomal function in microglia; reduction of pro-inflammatory cytokine expression; enhancement of anti-inflammatory gene expression; promotion of microglial transition from pro-inflammatory (M1) to anti-inflammatory (M2) phenotype	Neuroinflammation	(148)
*Bacteroides fragilis*	Production of PSA; restoration of intestinal barrier integrity; restoration of gut microbiota	UC	(32, 63, 149)
*Prevotella copri*	Potential enhancement of insulin sensitivity	Metabolic Syndrome	(150, 151)

#### 4.2.1 GABA

As the principal inhibitory neurotransmitter in the CNS, GABA can also be synthesized by commensal bacteria such as *Lactobacillus*, *Bifidobacterium*, and *Bacteroides*. Beyond regulating neuronal excitability and anxiety-related behaviors, microbial GABA exerts direct immunoregulatory effects. Engagement of GABA-A receptors on T cells and macrophages dampens pro-inflammatory cytokine production (IL-6, IL-1β) and restrains T-cell proliferation, thereby attenuating systemic inflammation and autoimmune responses (152). In addition, GABA promotes Treg differentiation and biases microglia toward an anti-inflammatory phenotype, underscoring its dual role in maintaining immune tolerance and neural homeostasis.

#### 4.2.2 5-HT

Over 90% of the body’s 5-HT is synthesized in the gut by enterochromaffin (EC) cells, which convert dietary L-tryptophan into 5-HT through the sequential actions of tryptophan hydroxylase 1 (TPH1) and aromatic L-amino acid decarboxylase (153). Certain intestinal bacteria, such as *Bifidobacterium*, *Lactobacillus*, and *Clostridium* species, possess tryptophan decarboxylase activity that enables them to directly convert dietary tryptophan into tryptamine or 5-hydroxytryptophan (154). These intermediates can then be absorbed by EC cells and further decarboxylated to complete 5-HT biosynthesis—essentially acting as a “pre-processing” step for serotonin precursors. In addition, microbial metabolites such as SCFAs can activate free fatty acid receptors 2 and 3 on EC cells, thereby upregulating TPH1 expression and enhancing tetrahydrobiopterin availability, which collectively increase 5-HT production (53). Other microbial products—including secondary bile acids (155), indole derivatives (153), and various cofactors—along with immuno-inflammatory modulation (156), can further activate TPH1 or L-amino acid decarboxylase activity, promoting serotonin synthesis within EC cells. SCFAs (53). Subsequently, this gut-derived 5-HT mediates key functions like propulsive motility by activating local receptors including the 5-HT4 receptor. Peripheral 5-HT influences immune function by acting on a broad spectrum of receptors (5-HT1A, 5-HT2A, 5-HT3, 5-HT4, 5-HT7) expressed on dendritic cells, T cells, and macrophages, thereby regulating cytokine secretion and T-cell fate decisions (157). In the CNS, 5-HT derived from microbial precursors directly influences microglial activity and synaptic remodeling, evidenced by suppressing pro-inflammatory cytokines (e.g., TNF-α, IL-1β, IL-6) and modulating the phagolysosomal compartment, while concurrently enhancing markers of synaptic plasticity such as BDNF and promoting dendritic spine complexity (2). Altered microbial control of serotonergic pathways has been linked to both neuroinflammation and psychiatric disease (114).

#### 4.2.3 Dopamine

Gut microbiota, including genera such as *Bacillus*, *Escherichia*, and *Enterococcus*, can directly synthesize dopamine or convert dietary precursors like the amino acid tyrosine and L-dopa into dopamine, influencing local gut signalling and contributing to systemic dopaminergic pools (158). Immune cells express dopamine receptors (DRD1–DRD5), allowing dopamine to modulate both innate and adaptive responses. Low-dose signaling through these receptors generally promotes anti-inflammatory outcomes, including reduced T-cell activation and diminished release of cytokines such as TNF-α and IFN-γ (159). In the CNS, microbial-derived dopamine precursors contribute to dopaminergic tone, influencing motor control, reward processing, and motivation. Perturbations in this axis have been implicated in Parkinson’s disease, depression, and inflammatory disorders (137).

#### 4.2.4 ACh

Certain commensal taxa, including *Lactobacillus plantarum*, produce Ach, a neurotransmitter central to both peripheral and central physiology (138). In the periphery, ACh is a critical mediator of the vagus nerve–driven “cholinergic anti-inflammatory pathway.” By engaging nicotinic and muscarinic receptors on macrophages, ACh suppresses the release of pro-inflammatory cytokines (TNF-α, IL-1β), thereby limiting systemic inflammation and protecting against septic shock. Within the CNS, cholinergic signaling is essential for learning, memory, and attention, and microbial modulation of this pathway may provide a mechanistic link between gut homeostasis, cognition, and neuroimmune balance (139).

#### 4.2.5 SCFAs

SCFAs—primarily acetate, propionate, and butyrate—are generated by microbial fermentation of dietary fibers such as resistant starch and inulin. Butyrate exhibits potent anti-inflammatory effects by inhibiting NF-κB activation in intestinal epithelial cells, suppressing pro-inflammatory cytokines including IL-6, IL-1β, and TNF-α (160). Concurrently, butyrate functions as a histone deacetylase inhibitor, promoting transcription of anti-inflammatory genes (133). *In vivo*, butyrate enhances epithelial barrier integrity by stimulating mucin production, inducing goblet cell differentiation, and promoting epithelial turnover, thereby limiting translocation of inflammatory stimuli (133).

SCFAs modulate both innate and adaptive immunity via G-protein-coupled receptors (GPR41, GPR43, GPR109A) expressed on neutrophils, dendritic cells, and T cells, influencing chemotaxis, phagocytosis, and cytokine secretion (161). Activation of GPR43 on neutrophils enhances reactive oxygen species production and pathogen clearance, while GPR109A signaling in dendritic cells increases retinoic acid synthesis, promoting a tolerogenic phenotype (162). Notably, SCFAs facilitate differentiation of peripheral Tregs, reinforcing immune tolerance and preventing autoimmunity (163).

Metabolically, SCFAs serve as energy substrates for colonocytes and immune cells. Butyrate undergoes β-oxidation within intestinal epithelial mitochondria, supporting ATP production and epithelial homeostasis (164). In T cells, SCFAs influence metabolic reprogramming by elevating intracellular acetyl-CoA and activating AMP-activated protein kinase, which favors oxidative phosphorylation over glycolysis. This shift promotes differentiation of Tregs and memory T cells while restraining pro-inflammatory Th1 and Th17 subsets (164). Furthermore, SCFAs suppress mTOR signaling, reducing inflammatory T cell proliferation and enhancing anti-inflammatory responses (165).

#### 4.2.6 Tryptophan metabolites

Tryptophan metabolism by gut microbes produces immunologically active compounds including indole derivatives (e.g., indole-3-acetic acid, indole [3, 2-b] carbazole), kynurenine (KYN), and downstream metabolites such as 3-hydroxykynurenine (166). Many act via the aryl AHR, a ligand-activated transcription factor broadly expressed in immune and epithelial cells (167). Upon ligand binding, AHR translocates to the nucleus, modulating gene expression linked to immune cell development and cytokine production. Commensal-derived indole derivatives like ICZ and FICZ activate AHR to suppress Th17 polarization and expand Tregs, thereby attenuating mucosal inflammation (136). Concurrently, AHR signaling regulates dendritic cell and macrophage maturation and function, fine-tuning antigen presentation and maintaining immune balance (136).

KYN pathway metabolites also influence adaptive immunity. KYN and 3-HK, endogenous AHR ligands, promote Foxp3 expression, facilitating Treg differentiation and creating an immunosuppressive environment (168). These tryptophan metabolites form a critical communication axis integrating microbial composition, metabolic output, and host inflammatory status. Thus, microbial metabolites such as SCFAs and tryptophan derivatives represent essential molecular mediators within the gut-immune axis, modulating immune cell programming and metabolic pathways with significant therapeutic implications.

Collectively, these microbiota-derived neurotransmitters exemplify the biochemical integration of microbial metabolism with host neuroimmune regulation. They not only facilitate direct communication between the gut and the brain but also calibrate immune responses, thereby influencing susceptibility to neuroinflammatory, autoimmune, and psychiatric disorders. Targeting the synthesis, reception, or degradation of these microbial neurotransmitters offers promising therapeutic avenues for diseases characterized by neuroimmune dysregulation.

## 5 Therapeutic implications and microbiota-targeted interventions

Elucidation of the gut–brain–immune axis provides a compelling rationale for microbiota-targeted therapies in neuroimmune disorders. Current strategies aim to reverse dysbiosis and restore homeostasis mainly through two primary approaches:

- **Microbiota-targeted supplementation.** This includes the use of prebiotics, probiotics, engineered live biotherapeutic products (LBPs), and postbiotics. Prebiotic fibers (e.g., inulin) selectively promote beneficial commensals and enhance production of immunomodulatory metabolites such as SCFAs (169, 170). Defined probiotics or LBPs (e.g., *Faecalibacterium prausnitzii*, Clostridium clusters IV and XIVa, and specific *Lactobacillus* or *Bifidobacterium* strains) reintroduce keystone taxa to enhance SCFA production, promote Treg differentiation (171, 172), strengthen gut integrity (173), and modulate neuroactive signaling (e.g., GABAergic pathways) (115). Alternatively, direct administration of microbial metabolites (postbiotics) such as SCFAs (e.g., sodium butyrate) or tryptophan derivatives (e.g., indole-3-propionic acid) offers a non-living strategy to directly influence host immunity and barrier function (109, 174).
- **Ecological Restoration via FMT.** FMT seeks to holistically restore a healthy microbial community, thereby concurrently improving metabolic output, epithelial barrier function, and immune regulation. Although FMT is most established in gastrointestinal conditions, preclinical evidence supports its potential to ameliorate neuroinflammatory and behavioral phenotypes in models of MS and AD (65, 70).

Clinical implementation of these interventions faces challenges, including microbiota heterogeneity, limited engraftment efficiency of biotherapeutics, and interactions with conventional immunotherapies. Future efforts should emphasize personalized strategies based on deep phenotyping of host–microbiota interactions to enable precision modulation of neuroimmune activity.

## 6 Conclusion and future perspectives

The intricate interplay among the gut microbiota, immune system, and CNS—often framed as the “gut–brain–immune axis”—is increasingly recognized as a central regulatory network governing human health and disease. Gut microbes not only sustain intestinal homeostasis but also engage in dynamic, bidirectional communication with the brain through neuroactive metabolites, microbial signaling molecules, and immune modulation. This complex ecosystem profoundly influences the pathogenesis of neurodegenerative diseases, autoimmune disorders, and metabolic syndromes.

Recent advances have illuminated pivotal roles for microbial metabolites such as SCFAs and tryptophan derivatives in orchestrating host immunity and neuronal function. Yet, critical mechanistic questions remain unresolved. In particular, the molecular integration of microbiota-derived signals in immune cell programming, BBB integrity, and neuroinflammatory pathways requires deeper elucidation. Although microbial dysbiosis holds promise as a biomarker for disease diagnosis, its predictive accuracy for early detection or clinical progression mandates validation through comprehensive, longitudinal cohort studies.

Moving forward, research should emphasize high-resolution dissection of host–microbe interactions using single-cell and spatial transcriptomics, paired with functional validation in GF animal models and human organoid systems. Such integrative approaches have the potential to reveal novel microbial effectors and host pathways that drive disease onset and progression. Concurrently, development of precision microbiome-based diagnostics and therapeutics—including engineered probiotics, tailored microbial consortia, and bioactive postbiotics—will be essential to achieve individualized modulation of immune and neural functions based on personal microbiota and immune profiles.

Moreover, the gut microbiome exhibits remarkable plasticity, responding dynamically to environmental factors such as diet, lifestyle, antibiotics, and stress. Deciphering how these external influences reshape microbial communities and their functional outputs will be critical for designing sustainable, non-invasive interventions. Integrative multi-omics and systems biology frameworks will play an indispensable role in capturing the temporal and physiological complexity of host–microbiota crosstalk.

In summary, the gut microbiota represents a frontier of biomedicine with transformative potential to advance immune regulation and neuroprotection. Continued interdisciplinary efforts promise to uncover novel biomarkers, therapeutic targets, and personalized strategies that will redefine prevention, diagnosis, and treatment paradigms for immune-mediated and neurodegenerative diseases.
